# Application of essential oils and polyphenols as natural antimicrobial agents in postharvest treatments: Advances and challenges

**DOI:** 10.1002/fsn3.1437

**Published:** 2020-05-16

**Authors:** Laura Maryoris Aguilar‐Veloz, Montserrat Calderón‐Santoyo, Yuliana Vázquez González, Juan Arturo Ragazzo‐Sánchez

**Affiliations:** ^1^ Laboratorio Integral de Investigación en Alimentos Tecnológico Nacional de México ‐ Instituto Tecnológico de Tepic Tepic México

**Keywords:** essential oil, natural antimicrobial agents, polyphenols, postharvest

## Abstract

The use of natural antimicrobial agents is an attractive ecological alternative to the synthetic fungicides applied to control pathogens during postharvest. In order to improve industrial production systems, postharvest research has evolved toward integration with science and technology aspects. Thus, the present review aims to draw attention to the achieved advances and challenges must be overcome, to promote application of essential oils and polyphenols as antimicrobial agents, against phytopathogens and foodborne microorganisms during postharvest. Besides that, it attempts to highlight the use of coating and encapsulation techniques as emerging methods that improve their effectiveness. The integral knowledge about the vegetable systems, molecular mechanisms of pathogens and mechanisms of these substances would ensure more efficient in vitro and in vivo experiences. Finally, the cost‐benefit, toxicity, and ecotoxicity evaluation will be guaranteed the successful implementation and commercialization of these technologies, as a sustainable alternative to minimize production losses of vegetable commodities.

## Graphical abstract

Advances and challenges must be overcome to promote application of essential oils and polyphenols as antimicrobial agents. Use of coating and encapsulation techniques as emerging methods that improve their effectiveness

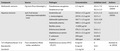

## 1 INTRODUCTION

The sustainability of the agroalimentary industry it is a global need, and in developing countries, it faces more risks, due to technological disadvantages and climate change phenomena. Particularly, in the Latin America and the Caribbean area great attention is drawn to the food losses and waste dimension, mainly in postharvest processes. Only for fresh fruit and vegetables, the estimated losses are around 55% (FAO, 2019; Eguillor, 2019). The pathogenic fungi and bacteria are the fundamental responsible of the quality and nutrient composition deterioration and mycotoxin contamination of products, affecting their conservation, useful life, and commercial value (Arias & Toledo, 2018; Cortés‐Higareda, Ramos‐García, Correa‐Pacheco, & Río, 2019; Sandoval‐Contreras, Villarruel‐ López, Sierra‐ Beltrán, & Torres‐Vitela, 2017; Sanzani, Reverberi, & Geisen, 2016).

Considering this background, many efforts are currently dedicated to develop research works related to the control of postharvest diseases. Besides the influence of the environmental factors (temperature, relative humidity, light, oxygen concentration, etc.), the attention has been focused to study the metabolic processes of pathogens and those that take place in fruit or vegetables during respiration and transpiration (Cortés‐Higareda et al., 2019; Wenchao, Zhang, & Bhandari, 2019).

Taking into account, that postharvest process is focused on food safety and food properties conservation (nutritional, taste, aroma, and good appearance), the study about disinfection of foodborne bacteria, which threaten the health of consumers, is among the aspects that must be considered during postharvest research also (Gómez‐López, 2012; Leyva et al., 2017; Meireles, Giaouris, & Simıes, 2016).The application of chemical microbicides continues to be the main form of pathogens control in different contexts. However, in the last decade, their intensive use has caused great concern about important issues related to environmental and human health (Arias & Toledo, 2018; García‐Robles, Medina‐Rodríguez, Mercado‐Ruiz, & Báez‐Sañudo, 2017; Mari, Bautista‐Baños, & Sivakumar, 2016; Palou, Ali, Fallik, & Romanazzi, 2016). This situation is inducing to reinforce the regulations regarding the allowed maximum residue limit in the products ( Regulation (EU) No 528/2012).

In order to ensure safe and quality food, different physical, chemical, and biological methods are being developed (De la Vega, Cañarejo, & Pinto, 2017; Dobry, Wisniewski, Teixido, Spadaro, & Jijakli, 2016; Franco‐Vega, Palou, & López‐Malo, 2012; Gómez‐López, 2012; Romanazzi et. al., 2016; Sivakumar & Bautista‐Baños, 2014). Among these, the biological methods have received great attention as a viable and safer alternative. In this sense, the antagonistic microorganisms as biological control agents have been investigated in laboratory, semi‐commercial, and commercial settings (Bautista‐Baños, 2006; Droby et al., 2016; González‐Estrada, Carvajal‐Millán, Ragazzo‐Sánchez, Bautista‐Rosales, & Calderón‐Santoyo, 2017; Parafati, Vitale, Resstucia, & Cirvirelly, 2016).

By the other hand, plant extracts containing compounds with antimicrobial properties aroused the most interest also (Mari et al., 2016; Palou et al., 2016; Pino, Sánchez, & Rojas, 2013). Thus, some vegetable volatile organic compounds (VOCs), such as aldehydes (acetaldehyde, 2‐E‐hexenal and benzaldehyde), alcohols, acetic acid, isothiocyanates, and mainly essential oils (EOs) have received consideration (Kumar & Kudachikar, 2018; Mari et al., 2016; Palou et al., 2016; Sivakumar & Bautista‐Baños, 2014; Torrenegra, Nerlis, Pájaro, & León‐Méndez, 2017; Yilmaz, Ermis, & Boyraz, 2016). In addition, other nonvolatile compounds, including plant extracts, as peptides and small proteins (Mari et al., 2016; Palou et al., 2016), chitosan and *Aloe vera* biopolymers (Bautista‐Baños, Hernández‐López, Bósquez‐Molina, & Wilson, 2003; Palou et al., 2016) and phenolic compounds (PPhs) have also been studied (Cushnie & Lamb, 2005; Loizzo et al., 2010; Pagnussatt et al., 2013; Pino et al., 2013; Rodríguez‐Maturino et al., 2015; Ruiz‐Barbai, Ríos‐Sánchez, Fedriani‐Irisol, Oliasi, & Rios, l., Jiménez‐Díaz, R., 1990; Sarkar & Shetty, 2014). In this sense, the PPhs must be considered very attractive compounds because in fact they have been reported as responsible of the antimicrobial activity of several EOs (Guarda, Rubilar, Miltz, & Galotto, 2011).

Taking into account the importance of the research and techno‐industrial strategies development, related with implementation of postharvest conservation technologies, the present work aims to update the state of the art about the study of EOs and PPhs as antimicrobial agents with potential use in postharvest field. Besides that, some challenges to be overcome for the industrial applications and commercialization of these antimicrobials in postharvest treatments should be evaluated.

## 2 ESSENTIAL OILS AND POLYPHENOLS AS NATURAL ANTIMICROBIAL AGENTS

Among the plant extracts that favor the development of efficient management strategies against microbial contamination, EOs have been the most investigated compounds among other antimicrobials (Kumar & Kudachikar, 2018; Mary et al., 2016; Rao, Chen, & McClement, 2019).

## 3 ESSENTIAL OILS

The EOs are included among VOCs that are produced as secondary metabolites by plants and microorganisms. They have been extensively studied as aromas and flavoring substances, appreciated for food applications (Bosquez‐Molina, Bautista‐Baños, Morales‐López et al., 2009). Furthermore, they have received a great attention for their antimicrobial action, useful during postharvest process (Kumar & Kudachikar, 2018; Mari et al., 2016). As GRAS (Generally Considered as Safe) substances, they have been applied in the agro‐food market, and some of them such as thyme (*Thymus vulgaris *L.), clove (*Syzygium aromaticum* L. Merr. & L.M.Perry), and mint oil (*Mentha rotundifolia* L. Huds) have included in the pesticide database by the European Commission (http://ec.europa.eu/food/plant/plaguicidas/eu-pesticidas-database-redirect/index_en.htm) (Mari et al., 2016).

The extraction of EOs is possible from the plants that belong to several typical botanical orders, using different parts of the tree (bark and wood, flowers, fruit, peel, leaves, roots, and exudates) (Kumari et al., 2014; Mari et al., 2016). EOs mainly contain volatile terpenoids (monoterpenoids and sesquiterpenoids) with different characteristic functional groups (Rao, Chen, & McClement 2019). At least 100 different compounds (aldehydes, phenols, oxides, esters, ketones, alcohols, and terpenes) may be present in each EO. They are unstable compounds, because of their volatility, photo‐, and temperature sensitivity (Troncoso‐Rojas et al., 2013), properties that must be considered during extraction procedures by classical or emerging techniques (Al‐Mamoori & Al‐Janabi, 2018; Azmir et al., 2013; Bosquez‐Molina et al., 2009; Chen, Hu., Yan‐Dong, & Hanhua‐Liang, Y., 2016; Fernández, Minuti, Visinoni, Cravotto, & Chemat, 2013).

Many reports have demonstrated that EOs and their chemical constituents have a broad spectrum of antimicrobial activities mainly against foodborne pathogens (Bosquez–Molina, Ronquillo–de Jesús, Bautista–Baños, Verde–Calvo, & Morales–López, 2010; Pino et al., 2013). For instance, the inhibitory action of EOs obtained from different Citrus species as mandarine (*Citrus reticulata* Blanco), lemon (*Citrus aurantifolia* (Christm.) Swingle), and orange (*Citrus sinensis* L.*)*, against some bacterial strains (*Escherichia coli*,* Staphylococcus aureus*,* Bacillus cereus*) and yeast (*Candida albican)* was demonstrated by Fisher and Phillips (2008). In this sense, Espina et al. (2011) showed their antimicrobial activity also, against *Enterococcus faecium*,* Pseudomonas aeruginosa*, and *Salmonella enterica.* A low minimal inhibitory concentration (MIC) of 0.2 μl/ml, in combination with a mild heat treatment (54 °C/10 min) showed synergistic lethal effect. Besides that, Torrenegra et al. (2017) during in vitro evaluation of these EOs against ATCC strains of *S. aureus*, *Staphylococcus epidermidis*, *Klebsiella pneumoniae*,* P. aeruginosa*, and* E. coli*, found strong activities with values of MIC ≥ 600 mg/ml. In general, due to its high content of monoterpenes, in particular limonene, these extracts have been considered very promising for postharvest applications, in direct oil and vapor forms.

Recently, both *Coridothymus capitatus* L. Rchb.f. and *Origanum syriacum* L. oils showed an antifungal activity (MIC ≤ 0.625 μl/ml). Interestingly, all foodborne pathogens tested were sensitive to first oil and two of them, *S. aureus* and *Listeria monocytogenes*, to second one (MIC ≤ 1.25 μl/ml) while the beneficial food‐related bacteria (*Lactobacillus *sp.) were not affected (MIC ± 10 μl/ml). Finally, *Cinnamomum zeylanicum* Blume oil showed a broader antimicrobial activity on all microorganisms analyzed.

Concerning the phytopathogenic bacteria, some reports have been founded about their sensitivity to EOs. Thus, the antimicrobial activity of 19 EOs was screened by Borboa‐Flores et al.. (2010) in order to control *Clavibacter michiganensis* subspecies *michiganensis*, which causes serious losses to tomato crops. Six of them were selected by their bactericidal activity at dilutions 1:1, 1:5, and 1:10. The best results were shown by *Lippia palmeri* S. Wats,* T. vulgaris *L., and *C. zeylanicum* J. Presl. The variance analysis revealed significant difference between these EOs, their concentrations as well as interaction between both two parameters in the bacterial growth inhibition.

Furthermore, oil of *Piper marginatum* Jacq. and its components showed a strong antibacterial activity with minimal bactericidal concentration (MBC) and MIC of 0.25 and 0.12 mg/ml, respectively, against *Xanthomonas albilineans* (Ashby) Dawson and *Xanthomonas campestris* pv. c*ampestris* (Pammel) Dowson. The principal component responsible for the biological activity was safrole (Sánchez, Correa & Y. y Pino, O., 2012a). After that, a strong antibacterial activity of *Ocimum basilicum* L. and *Ocimum basilicum* var. *Genovese* EOs against *C. michiganensis* subsp *michiganensis*, *X. albilineans* (Ashby) Dowson, *Streptococcus suis*, and *K. pneumonia* was demonstrated also (Sánchez, Pino, et al., (2012b).

On the other hand, different EOs such as *T. vulgaris L*.,* P. anisum* L*.*,* C. sinensis* L*. Osbeck.*,* O. basilicum* L*.*,* Curcuma longa* L*.*,* Ruta chalepensis* L*.*, and *Melaleuca quinquenervia* (Cav) S.T. Blake were evaluated against *Pectobacteriuum carotovorum* subspecies, which affect potatoes production and other crops of economic importance. Among them, the thyme oil showed stronger antibacterial activity (MIC = MBC =0.1%) (Rojas et al., 2014).

Regarding their fungicidal properties, different EOs have been evaluated against pathogenic fungi. Among them, more frequently used EOs are those from oregano (*Origanum vulgare* L*.*), basil (*Ocimum selloi* L*.*), savory (*Satureja hortensi*s L.), dill (*Anethum graveolens* L*.*), laurel (*Lauris nobilis* L*.*), and thyme (*T. vulgaris *L.). The last one has been recognized as very effective, because of this action against a great variety of pathogens, at 200–300 μg/ml: *Fusarium *sp. (Barrera & García, 2008), *Aspergillus* (Pekmezovic, Rajkovic, Barac, Senerovic, & Arsic, 2015); *Alternaria*,* Colletotrichum* and *Botrytis* (Mari et al., 2016). For instance, *Fusarium* sp. showed total inhibition with *T. vulgaris *L., while *C. zeylanicum*, J. Presl, *S. aromaticum* L., and *Teloxys ambrosioides* L. oils exhibited their action at higher doses, and *Allium sativum* L*.*,* C. aurantifolia* (Christm.) Swingle,* R. chalepensis* L*.*,* Mentha piperita* L., and *Eucalyptus globulus* L. oils had no activity. Concerning fungus *Botrytis cinerea*, during screening of some EOs, oils of the different plants were found to exhibit absolute fungitoxic activity, greater in comparison with some prevalent synthetic fungicides, in vitro and in vivo tests (Tripathi, Dubey, & Shukla, 2008).

Special attention has been dedicated to *Colletotrichum gleosporioides* because it is a cause of anthracnose, which provokes great losses in fruit production (Bosquez–Molina et al., 2010; Kumar & Kudachikar, 2018; Yilmaz et al., 2016). During in vitro and in vivo investigations with different EOs against anthracnose in apple was demonstrated that their fungicidal activity depends on the content of the metabolites carvacrol, thymol, cimeno, pinene, linalool, and ether acetate. However, their activity depends on the synergic action with the other minority components.

Recently, research has been focused on characterization of the specific major EOs extracts components present in different oils in relation to their antibacterial and antioxidant properties. Indeed, most of the constituents of common EOs that exhibit high antimicrobial efficacy are phenols, followed by oxygenated terpenoids. Thymol, eugenol, and mainly carvacrol, from thyme, clove, cinnamon, and oregano oils, respectively, ensure a broad spectrum of antimicrobial efficacy against Gram‐negative, Gram‐positive bacteria, and some pathogenic fungi; stronger than terpenes and other compounds (Guarda et al., 2011; Rao et al., 2019).

## 4 POLYPHENOLS

The PPhs are secondary metabolites widely distributed in plants, where they fulfill various functions related to growth and reproduction, allelopathy, and protection against pathogens, predators, diseases, and UV radiation. Usually, they do not have a significant toxic effect depending on their concentration and gallic acid content (Paredes‐López, Cervantes‐Ceja, Vigna‐Pérez, & Hernández‐Pérez, 2010).

PPhs refer to a group of chemical compounds that consist of hydroxyl groups bonded directly to an aromatic carbon. They have different structural and physicochemical properties and possess a diverse and heterogeneous reactivity and lability to different environmental factors such as pH, temperature, oxygen, light, etc. (Bakowska‐Barczak & Kolodziejczyk, 2011; Manchón, 2013). Usually, they are classified as flavonoids and nonflavonoids compounds. Dozens of different flavonoids may be present in the same plant species. Some of them are conjugated with various sugars and further distinguished by the number and arrangement of the hydroxyl groups, and degree of alkylation and glycosylation (Manchón, 2013).

Many investigations about the PPhs from different sources have been oriented to the study of their antioxidant properties, attractive for food industry applications (Paredes‐López et al., 2010; Ruiz‐Barbai et al., 1990). However, in last decade an increased interest has been showed in relation to their antimicrobial properties. They are obtained not only from the fruit, but also from other parts of the plant: root, stem, leaves, bark, etc. (Azmir et al., 2013; Ojwang, Muge, Mbatia, Mwanza, & Ogoyi, 2017; Sarkar & Shetty, 2014). Interestingly, many research works related to PPhs and other phytochemicals have recently been carried out from leaves of different specimens belonging to the *Moraceae* family: *Artocarpus* L*.* (Loizzo et al., 2010; Jagtap & Bapat, 2010; Ojwang et al., 2017), Mulberry (*Morus *spp.), as *Morus alba* L. (Khan et al., 2013), and from different *Ficus *spp. (Awolola, Chenia, Koorbanaly, & Baijnath, 2014; Salem, Salem, Camacho, & Hayssam, 2013; Usman, Abdulrahman, & Usman, 2009) (Table 1).

**Table 1:** Antimicrobial effect of plant phenolic extracts on foodborne bacteria (in vitro)

Compound	Source	Pathogen	Concentration	Inhibition (mm)	Authors
Methanolic extracts	Fig tree (*Ficus thonningii* L*.*)	*Pseudomonas aeruginosa*	2.5 mg/ml	33.3 ± 7.3	Usman et al. (2009)
		*Streptococcus *spp		32.3 ± 2.5	
Aqueous extracts	Jack fruit leaves (*Artocarpus heterophyllus* Lam)	*Escherichia coli*	343.6 ± 4.5 µg/ml	11.3 ± 1.5	Loizzo et al. (2010)
		*Listeria monocytogenes*	237.8 ± 2.2 µg/ml	12.5 ± 1	
		*Salmonella typhimurium*	467.7 ± 1.9 µg/ml	13.0 ± 0.8	
		*Salmonella enterica*	488.1 ± 3.4 µg/ml	7.5 ± 0.7	
		*Bacillus cereus*	*>*1,000 µg/ml	_	
		*Enteroccocus faecalis*	276.2 ± 3.1 µg/ml	14.0 ± 1	
		*Staphylococcus aureus*	245.2 ± 2.2 µg/ml	15.0 ± 1	
		*Fusarium culmorum*	300 ppm	31.81%	
		*Fusarium solani*	300 ppm	45.58%	
7,4'‐trihydroxyflavan−3‐ol	*Ficus sansibarica* Warb. subsp.* sansibarica*	*Staphylococcus aureus* ATCC 29,213	8 mg/ml	0	Awolala et. al. (2014)
Epicatechin			8 mg/ml	13.0	
Isovitexin			1.6 mg/ml	0	

Phenolic extracts of these plants have been shown a broad spectrum of antimicrobial activity mainly against foodborne microorganisms (Table 1). For instance, *Ficus thonningii* Blume extracts inhibited the growth of *P. aeruginosa* and *Streptococcus spp*. among other pathogens (Usman et al., 2009), while *Ficus sansibarica* Warb. subsp.* sansibarica* extracts, rich in triterpenes and flavonoids components, showed antibacterial action against *S. aureus* and antibiofilm action (Awolola et al., 2014) (Table 1). In general, a broad range of concentrations of different extracts has been used but in the majority of papers the authors report values of growth inhibition less than 50%, results that are must be improved in order to obtain a good efficient during in vivo tests.

Regarding jackfruit tree, Loizzo et al. (2010) reported antibacterial activity against *E. coli*, *L. monocytogenes*, *Salmonella typhimurium*,* Salmonella enterica*,* B. cereus*,* Enteroccocus faecalis*, and* S. aureus* and antioxidant capacity of PPhs from leaves, extracted by different solvents. Aqueous extracts with concentration of 237–1,000 µg/ml caused 7.5–15.0 mm growth inhibition haloes.

Concerning phytopathogenic bacteria, Ramírez‐Reyes et al. (2015) observed a growth inhibition of *Pseudomonas carotovorum* by the action of floral ethanolic extracts of *Magnolia schiedean*, which was attributed to the presence of phenols among other active compounds.

For phytopathogenic fungi, satisfactory effects have been reported with some natural extracts mainly for *Alternaria alternata* and *Fusarium *sp. among other species. Results also show a high variability in relation to concentration and inhibitory capacity of evaluated substances (Table 2). Among various plant species tested, aqueous extracts of leaves of papaya (*Carica papaya* L.) and custard apple (*Annona reticulate* L.) showed important fungistatic effect against *Rhizopus stolonifer *and* C. gloeosporioides* in ciruela (*Spondias purpurea* L.) and mango (*Mangifera indica* L.) during fruit storage (Bautista‐Baños et al., 2003). Similarly, effect of bee propolis on different fungal species: *A. alternata*,* Aspergillus niger*,* Aspergillus parasiticus*,* B. cinerea*,* Fusarium oxysporum* f. sp. *melonis*, and *Penicillium digitatum* has been reported. After that, Ojeda‐Contreras, Hernández‐Martínez, Domínguez, and Mercado (2008) evaluated the effect of caffeic acid phenethyl ester, a component of propolis, to control *A. alternata* infecting tomato fruit. They concluded that it has a superior inhibitory action than commercial fungicide without negative effect on fruit quality.

**Table 2:** Antimicrobial effect of phenolic extracts of different origin on phytopathogenic fungi (in vitro)

Compound	Source	Pathogen	Concentration	Inhibition (%)		Authors
Caffeic acid phenethyl ester	Propolis	*A. alternate* from tomato (*Solanum lycopersicum* l., Mill.)	80 µM	32.5		Ojeda‐Contreras et al. (2008)
			90 µM	30.6		
			100 µM	30.0		
Methanolic extracts	Cherimoya (*Annona cherimola* Mill.)	*Fusarium oxysporum*	2000–10,000 ppm	53–89.55		Ochoa‐Fuentes et al.. (2012)
		*Fusarium culmorum*	2000–10,000 ppm	78.35–89.62		
		*Fusarium solani*	2,000–10,000 ppm ppm	45.25–62.5		
	Cinnamon (*Cinnamomum zeylanicum* J.Presl)	*Fusarium oxysporum*	300 ppm	43.15		
		*Fusarium culmorum*	300 ppm	31.81		
		*Fusarium solani*	300 ppm	45.58		
Phenolic extract	*Spirulina* LEB−18	*Fusarium graminearum* barley and wheat	3%–8% (p/p)	50		Pagnussatt et al. (2013)
Phenolic extracts	Chiltepín (*Capsicum annum var. glabriusculum*)	*Alternaria alternate*	100 mg/ml	38.5^a^	92.0^b^	Rodríguez‐Maturino et al. (2015)
		*Fusarium oxysporum*		—a	85^b^	
Carotenoids		*A. alternate*		38.5^a^	85.3^b^	
		*Fusarium oxysporum*		20.3^a^	96.0^b^	

a % inhibition of mycelial growth.

b % inhibition of conidial germination.

On the other hand, Pagnussatt et al. (2013) assessed *Spirulina* LEB‐18 phenolic extract for its antifungal activity on strains of *Fusarium graminearum*, isolated from barley and wheat. They demonstrated that this extract reduced the growth rate of the toxigenic species investigated (Table 2). In this sense, during in vitro evaluation of antifungal activity of extracts of cherimoya (*Annona cherimola* Mill.), and cinnamon (*C. zeylanicum* J.Presl) both extracts showed inhibitory effects on the mycelial growth and sporulation of *Fusarium oxysporum*,* Fusarium culmorum*, and* Fusarium solani* strains also*.* Cinnamon extracts were effective to control *Fusarium* species in doses of 330–539 ppm, while the best control of the cherimoya extract was at 593 ppm against *F. culmorum*, and at higher doses from 2,060 to 2,571 ppm for other species (Ochoa‐Fuentes, Cerna‐ Chávez, Landeros‐Flores, Hernández‐Camacho & y Delgado‐Ortiz J.C., 2012).

Therefore, interesting antifungal effect was showed by phenolic and carotenoid extracts of “chiltepin” (*Capsicum annum var. Glabriusculum*) against *F. oxysporum* and *A. alternata* strains in relation to mycelial growth inhibition and conidial germination inhibition (Rodríguez‐Maturino et al., 2015) (Table 2).

## 5 MECHANISMS OF ACTION

Regarding the action mechanisms of EOs and PPhs as antimicrobial agents, it is important to consider that their effectiveness will depend not only on the concentration and chemical nature, but also on the susceptibility and concentration of the pathogen, and even of the microbial strain characteristics. This capacity will vary depending on the composition and nature of the microbial cell surface (Melgarejo & Postilla, 2011; Rao et al., 2019). Furthermore, it is important to know and understand the mechanisms of action of pathogens, depending on the characteristics of the fruit and environmental conditions (Barkai‐Golan, 2001; Basak & Guha, 2018; Rao et al., 2019; Sanzani et al., 2016; Sarkar & Shetty, 2014).

Four possible mechanisms of action have been defined for antimicrobial substances: interference in the cellular structure; interference in cellular biosynthesis; inhibition of the energy mechanism and multisite activity (Calvo & Martínez‐Martínez, 2009; Melgarejo & Postilla, 2011; Rodríguez‐Maturino et al., 2015). Besides that, the environmental factors such as temperature, pH, medium composition, etc. and contact time will play an important role, as well (Barkai‐Golan, 2001; Sandoval‐Contreras et al., 2017). In the case of EOs, due to their hydrophobic nature, they could penetrate through bacterial cell outer membranes and cytoplasmic membranes into the interior of cell and thus disintegrate its structures. It renders them more permeable, causing the leakage of cellular components or inactivating the enzymes responsible for the cell wall synthesis and provoking molecular changes in their structure. In addition, the EOs have antioxidant properties effective in retarding the process of lipid peroxidation and scavenging free radicals (Rodríguez‐García et al., 2016). In the same manner, these structural modifications take place in fungi and would cause morphological variations of the hyphae and inhibition of conidial germination (Cushnie & Lamb, 2005; Rao et al., 2019; Rodríguez‐García et al., 2016).

In general, EOs and PPhs may provoke a physical, chemical, or biochemical change in the microorganisms, and different constituents may operate by different mechanisms and may target different kinds of microbes, such as Gram‐positive and Gram‐negative bacteria, yeasts, or molds, because they differ in the composition of their cell membranes. Therefore, it is difficult to predict how susceptible pathogens are and why the susceptibility varies from strain to strain after applying these compounds.

It has been proved that some tree EOs inhibit respiration and also causing potassium ion leakage in both Gram‐positive bacteria and Gram‐negative bacteria, and the most of their constituents are phenols (thymol, eugenol, and carvacrol), followed by oxygenated terpenoids (Rao et al., 2019).

Regarding PPhs some mechanisms are reported: formation of complexes with soluble and extracellular proteins, generating a disruption of the fungal cell wall (Rodríguez‐Maturino et al., 2015); reduction of the decomposition of H_2_O_2_, which affect many pathogens since it becomes highly reactive oxygen species promoting oxidative damage; action as a proton exchanger, and the resulting collapse of proton motive force and depletion of adenosine triphosphate (ATP) eventually lead to cell death (Rao et al., 2019).

PPhs could inactivate the essential amino acid synthesis caused by interference in the reactions of phosphoenolpyruvate, erythrose‐4‐phosphate, and shikimic acid. This favors the production of tryptophan and decreases the production of phenylalanine or tyrosine, modifying the structure of some proteins essential for the formation of fungal appressorium structure (Pagnussatt et al., 2013).

It has been shown that PPhs act at the membrane level by modifying the polar heads of the lipid molecules. Some results demonstrated that their antimicrobial activity is associated with the presence and position of a free hydroxyl group bonded directly to a C6 aromatic ring as a system for electron delocalization, that favor their ability to modify the microbial cell membrane integrity. Furthermore, the hydroxyl group has a key role in the inactivation of microbial enzymes such as ATPase, histidine decarboxylase, amylase, and protease. Inhibition of ATPase may be important for cell death due to disturbed cellular respiration (Rao et al., 2019).

For instance, tannins are able to block the activity of catalase, provoking a lethal effect on some pathogens (Zhao & Drlica, 2014). Also, they could inhibit enzymes involved in ergosterol synthesis, the main component of the fungal cell membrane, reducing its intracellular content (Campoy & Adrio, 2017; Rao et al., 2019). Besides that, it is important to take into consideration that EOs and PPhs compounds also could induce fruit resistance processes to pathogenic microorganisms, as other natural compounds do (chitosan, fructooligosaccharides, and vegetable hormones), provoking a sequential reaction by activation of particular genes and biosynthesis of antimicrobial substances (Romanazzi et al., 2016).

## 6 USE OF COATINGS AND FILMS FOR NATURAL ANTIMICROBIALS AGENTS’ APPLICATION

In order to prevent postharvest diseases, the natural antimicrobial agents can be used in vapor or liquid systems, mixed with surfactants in immersion tanks during the packaging process or in wax formulations. In the practice, these compounds have been incorporated separately, in sachets, in packaging systems, during storage, transport or even marketing, depending on the fruit (Mari et al., 2016). But many reasons difficult their incorporation to the biological formulations: fast release, low solubility, low bioactivity, and lability against environment stresses (temperature, moisture, pH, oxygen light, etc.). The oxidative degradation may deteriorate these compounds leading to the generation of free radicals and development of unpleasant tastes and off‐odors. It results in an undesirable effect on shelf stability, sensory characteristics, and consumer acceptability of the products (Ariyarathna & Karunaratne, 2016; Gómez‐Mascaraque et al., 2016; Shishir, Xie, Sun, Zheng, & Chen, 2018; Sánchez et al., 2016).

Therefore, in order to guarantee effective applications of natural antimicrobial agents, it must be considered that their stability and bioactivity will depend on their physicochemical properties (structure, molecular mass, solubility, and reactivity) and agroindustrial application conditions (Kamil, Chen, & Blumberg, 2015; Rodríguez, Martín, Ruiz, & Clares, 2016; Shaaban, El‐Ghorab, & Shibamoto, 2012). Thus, during the last decades their application has been diversified through edible coatings and films development, because these technologies represents an opportunity of mitigation of environmental risks and improving of the food quality (Fernández, Echeverria, Mosquera, & Paz, 2017; Marín, Atarés, & Chiralt, 2017; Ponce, Roura, Valle, & Moreira, 2008; Ramos–García, Bautista‐Baños, & Estrada–Carrillo, 2010; Yuan, Chen, & Li, 2016).

These techniques combine the principles of physical, chemical, and biological methods and include the use of GRAS ingredients of different nature such as salts, peptides, small proteins, EOs, PPhs and other plant extracts (Aloui et al., 2014; Mari et al., 2016; Ponce et al., 2008), antagonistic microorganisms (González‐Estrada et al., 2017; Parafati et al., 2016), etc.

An edible coating creates a modified atmosphere by constituting a barrier to gases (O_2_, CO_2,_ and water vapor), reduces the speed of breathing and the weight loss during storage and transport, delaying the process of fruit senescence (Ramos–García et al., 2010). Furthermore, many biological materials including polysaccharides, proteins, lipids, and their mixtures have been employed, considering also the morphological and physiological characteristics of the products, their condition as climacteric or nonclimacteric fruit. Films properties (transparency, brightness, resistance, etc.) and effectiveness of antimicrobial agent action will depend on biopolymers and high biological value compounds (HBVC) characteristics (Marín et al., 2017; Yuan et al., 2016).

For instance, antifungal activity of some natural additives added to hydroxypropyl methylcellulose‐lipid edible coatings against *B. cinerea* and *A. alternata* was exhibited in vitro on cherry tomato (Fagundes, Pérez‐Gago, Monteiro, & Palou, 2013), while grapefruit EO or grapefruit seed extract containing in alginate coatings have been successfully used for grapes preservation (Aloui et al., 2014).

In the same way, chitosan edible films and coatings are promising systems to be used as EOs and PPhs carriers in postharvest applications, owing to their antioxidant and antimicrobial activities. This combination can provide improved additive or synergistic interactions, showing greater effectiveness against fungi and foodborne bacteria in food systems than pure films and coatings (Ávila‐Sosa et al., 2012; Yuan et al., 2016).

For instance, Bautista‐Baños et al. (2003) evaluated the fungicidal effect of chitosan (2.5%) and aqueous extracts of custard apple leaves, papaya leaves, and papaya seeds on the growth of *C. gloeosporioides in* papaya fruit. During in vitro test, the combination of 2.5% chitosan with all the extracts had an improved fungistatic effect, while in vivo studies, control of anthracnose disease was obtained with 1.5% chitosan. Furthermore, alterations in the hyphae and spore development of *F. oxysporum* sp. g*ladioli* and *R. stolonifera*, and lyses of *A*. *alternata* cells after treatment with chitosan and natural product mix, were observed (Bautista‐Baños et al., 2012). Besides that, Ávila‐Sosa et al. (2012) revealed the antifungal effects of edible films of chitosan, amaranth, and starch with Mexican oregano oil (0.5%) against *A. niger* an*d P. digitatum*.

In the complex host/antimicrobial compound/pathogen system, many biochemical processes can occur with different effects on biological activity of the antimicrobial compound. The pathogens would be less likely to develop resistance against the mixture of compounds. Furthermore, compounds present in the coatings may act as elicitors of resistance through different mechanisms mediated by the host tissue (Yuan et al., 2016). For instance, the combined coating could control the EO vapor diffusion and reduce the structural changes or loss of HBVC. The coatings, films, and microcapsules are more profitable because small volumes are applied, product contamination is avoided, and the prolonged action is guaranteed. The use of small volumes ensure effective conservation of the products without affecting their sensory properties (Cortés‐Higareda et al., 2019; Ezhilarasi, Karthik, Chhanwal, & Anandharamakrishnan, 2013; Kamil et al., 2015; Parafati et al., 2016; Wenchao et al., 2019).

Furthermore, from the commercial point of view, the implementation of new coating technologies does not generally require the acquisition of new equipment or space, since many fresh produce packinghouses already employ waxing equipment (Fernández et al., 2017; Mari et al., 2016). Besides that, a greater emphasis has been placed on the currently films development from new natural biopolymers, due to the growing demand for sustainable food production and opportunities to open new markets from nontraditional agricultural products and wastes. Recently, the use of conventional raw materials (tubers, rice, amaranth, and quinoa) has allowed to obtain films and coatings with good mechanical and barrier properties (Mari et al., 2016).

## 7 ENCAPSULATION OF NATURAL ANTIMICROBIAL COMPOUNDS

In postharvest processes as in food practice, applications of encapsulation methods have been linked with ensuring controlled releasing and avoiding undesirable structural and bioactivity changes of HBVC. Micro‐ and nano‐encapsulation are two major ways of this technology, able to improve product functionality. Recently, there have been found remarkable interest in development of these nano‐scale delivery systems for this compounds due to their advantages—high encapsulation efficiency and loading capacity, enhanced bioavailability, improved stability, sustained release profile, and masking undesirable flavors (Esfanjani & Jafari, 2016; Fathi, Martín, & McClements, 2014; Kamil et al., 2015; Liang et al., 2017; Maes, Bouquillon, & Fauconnier, 2019; Shishir et al., 2018; Yu et al., 2018).

Micro‐ and nanocapsules have been used as a functional ingredient in coatings, edible films, and formulations. Depending on compounds and their final application, different physical and chemical methods have been applying, using biocompatible and biodegradable polymeric materials, natural or synthetic, food grade or GRAS. They allow improving the physicochemical properties of the capsules (microstructure, thermal stability, solubility, hydrophobicity, zeta potential, etc.) in order to guarantee an adequate release behavior of the HBVC and their biological mechanism (Kamil et al., 2015; Kothalawala & Sivakumaran, 2018; Maes et al., 2019).

Among the used methods, reports mainly have been focused to coprecipitation (emulsion/ solvent evaporation), freeze drying and spray drying (Ballesteros, Ramírez, Orrego, Teixeira, & Mussatto, 2017; Çam, İçyer, & Erdoğan, 2014; Guarda et al., 2011; Ribeiro & Pedreira, 2018). During last years, satisfactory results with electrodynamic techniques have been reported. Nano‐capsules and natural nano‐carriers for phenolics have been developed using cyclodextrins, nano‐caseins, nano‐crystals, among other matrixes by electrospraying, electrospinning, and nano‐spraying (Drosou, Magdalini, Krokida, & Biliaderis, 2010; Esfanjani & Jafari, 2016; Hernández et al., 2015). It has shown that the technique, nature of the coating material and HBVC also greatly influenced the encapsulation capacity (Ballesteros et al., 2017).

Concerning the antimicrobial effectiveness of micro‐ and nanoparticles, some attractive results have been obtained (Table 3). An improvement in the antimicrobial action of monoterpenoid phenols, carvacrol, and thymol was observed when they were added as microcapsules in flexible plastic film coating (Guarda et al., 2011). In this case, microcapsules were prepared using emulsion of soybean oil in aqueous solution of arabic gum and tween 20. They showed a strong activity against several foodborne microorganisms: *E. coli O157: H7*,* S. aureus*,* L. innocua*,* S. cerevisiae*, and* A. niger*, but inhibited mainly the growth of mycelium. A significant activity for thymol and carvacrol with MIC of 125–250 ppm and 75–375 ppm, respectively, was observed, and highest synergism at 1:1 relation.

**Table 3:** Application of encapsulation techniques for improving effectiveness of natural antimicrobial compounds against different pathogens

Antimicrobial compound	Source	Encapsulating material/ method	Pathogen	Authors
Carvacrol and thymol	Oregano (*Origanum vulgare* L*.*)	Microcapsules (by emulsification of soybean oil in aqueous solution of Arabic gum + Tween 20) contained in chitosan flexible plastic film coating	*Escherichia coli* O157:H7 *Staphylococcus aureus* *L. innocua* *S. cerevisiae* *Aspergillus niger*	Guarda et al.(2011)
Extract of phytochemicals	Acerola *(Malpighia emarginata*; DC) guava (*Psidium guajava)* passion fruit (*Passiflora edulis* Sims)	PLGA nanoparticles (emulsion‐evaporation method)	*L. monocytogenes* Scott A. *E. coli* K12	Silva et al. (2014)
Extract of hydrophobic phytochemicals	Guavira (*Campomanesia xanthocarpa* O. Berg)	PLGA (emulsion‐evaporation method)	*L. innocua*	Pereira et al. (2015)
Phenolic extracts	Passion fruit (*Passiflora edulis* Sims)	PLGA (coprecipitation method)	*L. innocua*	Oliveira et al. (2017)
Polyphenols, carotenoids	Guavira (*Campomanesia* (*xanthocarpa* O. Berg)	PLGA (emulsion‐evaporation method)	*L. innocua*	Pereira et al. (2018)
eugenol	Litchi (*Litchi chinensis* Sonn.)	β‐cyclodextrins (method of saturated aqueous solution)	*Peronophythora litchii*	Gong et al. (2016)
(+) Catechin and (−)‐epicatechin	Green tea (*Camellia sinensis* (L.) Kuntze)	Hydroxypropyl‐β‐cyclodextrin (Isothermal titration calorimetry)	*–*	Liu et al. (2016)
Eugenol and carvacrol	Clove (*Eugenia caryophyllata* Thunb.) Mexican oregano (*Lippia berlandieri* Schauer)	β‐cyclodextrin (coprecipitation method).	*L. monocytogenes* ATCC 19114 *E. coli* ATCC 25922	Anaya‐Castro et al. (2017)
ácidos fenólicos y flavonoids	Xoconostle (*Opuntia oligacantha* C. F. Först)	Emulsion with essential oil of *Citrus sinensis*	*C. gloesporoides*	Solís‐Silva et al. (2018)
Polyphenols	propolis	Chitosan (1%) nanoparticles (by emulsification with glycerol and canola oil)	*Aspergillus flavus*	Cortés‐Higareda et al. (2019).

Among synthetic polymers, polylactates (PLA) and their polyethylene glycol derivatives (PLGA) have been used (Table 3). In this sense, nanoencapsulation and delivery of phytochemicals of tropical fruit by‐products was studied by Silva, Hill, Figueredo, and Gomes (2014). They demonstrated the advantages of the use of this matrix for antioxidant and antimicrobial applications. The same manner, nanoencapsulation of hydrophobic phytochemicals and PPhs of guabiroba fruit (*Campomanesia xanthocarpa* O. Berg) was achieved by Pereira, Hill, and Zambiazi (2015) and Pereira et al. (2018). Evaluation of capsules with these compounds against the high‐resistant Gram‐positive bacterium, *L. innocua*, showed a significantly greater growth inhibition in comparison with nonencapsulated compounds. Furthermore, capsules of polyphenolic extracts of passion fruit (*Passiflora edulis* Sims) in PLGA were obtained using the coprecipitation method (Oliveira, Ferreira, & Gomes, 2017). These authors observed 23.8%–79% efficiency and a greater antimicrobial action in comparison with the whole extracts (Table 3).

Therefore, β‐cyclodextrin (CD) and their derivative hydroxypropyl‐β‐cyclodextrin have been used successfully also for encapsulation of PPhs as eugenol and (+) catechin and (−)‐epicatechin, improving their stability (Table 3). In vitro assays showed inhibitory effect of complex CD–eugenol on of *Peronophythora litchii* colony growth in a concentration‐ and time‐dependent manner (MIC = 0.2 g), while i*n vivo* assays showed its impact on reduction of the decay index of treated fresh litchi fruit. After exposure to CD–EG, the surface of fungal hyphae and/or sporangiophores became wrinkled, with folds and breakage. Besides that, damage to cell walls and membrane structures was confirmed (Gong, Li, Chen et al., 2016). On the other hand, investigation about complexation of two isomers, (+)‐catechin and (−)‐epicatechin, with hydroxypropyl‐b‐cyclodextrin in Tris–HCl buffer solutions at pH 6.8–8.0 using isothermal titration calorimetry, was carried out. Stability study indicated that a complex with CD showed a stronger but different protection effect on isomers, depending on their molecular structure (Liu et al., 2016).

Besides that, Anaya‐Castro et al. (2017) evaluated encapsulation efficiency of clove and Mexican oregano EOs in β‐cyclodextrin matrix by determination of eugenol and carvacrol, respectively. The 4:96 ratios (clove EO/CD) gave the highest eugenol content and greatest microencapsulation efficiency; and the 8:92 and 12:88 ratios (Mexican oregano EO/CD) the highest carvacrol content. After that, antimicrobial activity of the complexes was tested against *L. monocytogenes* ATCC 19114 and *E. coli* ATCC 25922. Inclusion of the majority of biologically active clove and Mexican oregano EOs molecules, providing increased stability by reducing their volatility and preserving their biological properties was demonstrated.

All these results show the potentialities of nanoencapsulation of EOs and PPhs compounds (Liang et al. (2017). However, they have been developed mainly against foodborne microorganisms and for pharmaceutical and food applications. Concerning postharvest treatments, fewer works have been reported. For instance, a study of a nanoemulsion of xoconostle (*Opuntia oligacantha* C. F. Först) with EO of *C*. *sinensis*, demonstrated that it is an excellent source of phenolic acids and flavonoids, appropriated to significantly affect the *C. gloesporoides* growth. Nonencapsulated compounds showed minor values of the cell lysis haloes diameter in solid medium, in comparison with encapsulated ones (17.1 y 21.25 mm respectively) (Solís‐Silva et al., 2018). Respecting these results, emulsions are a probable approach to guarantee appropriate delivery systems of HBVC with maximum efficacy in the food systems. Nanoemulsions could contribute to increase their dispersibility improving their bioavailability and bioactivity (Basak & Guha, 2018; Donsi & Ferrari, 2016).

On the other hand, characterized nanostructured chitosan/propolis formulations demonstrated their inhibitory effect on the growth of *A. flavus*, its spore germination (97%), and aflatoxin production (100%) (Cortés‐Higareda et al., 2019).

## 8 CHALLENGES IN RESEARCH AND APPLICATION OF NATURAL ANTIMICROBIALS IN POSTHARVEST SYSTEMS

According to ethnobotanical studies in our context, there is great tropical plant biodiversity with possibilities of being used in postharvest and other agroindustrial processes, because they are sources of HBVC. However, the possibilities of their commercial scale production and practical applications are limited, first because of the current availability of highly effective, and cheaper conventional fungicides. Thus, this technology presents a major challenge in the coming years, because all the aspects related to it have not yet been clarified and the development capacities, knowhow about in situ processing good alternatives have not been guaranteed for local development (Mari et al., 2016).

Inconsistent results are often reported, depending on the type of crop or nature of disease or storage conditions. In some research studies, biological evaluation does not always correlate with chemical composition and identification of the main compounds (Pino et al., 2013). This has wide implications in terms of developing and commercializing this technology.

In order to standardize the commercial production of natural antimicrobials, multiple factors causing their high variability must be considered. They depend on (a) variety and cultivation conditions, influenced by the climatic factors (photoperiod, shade, temperature, etc.); the plant age (maturity) and agronomic practices (soils, time of planting and time of harvest, application of fertilizers, irrigation, etc.) (Kumari et al., 2014), (b) part of plant used in the biological evaluation (Loizzo et al., 2010; Ojwang et al., 2017), (c) experimental procedures for extraction and conservation (Manchón, 2013), and (d) methods of chemical characterization (Azmir et al., 2013).

On the other hand, it is indispensable to increase in vivo practices, under semi‐controlled or real production conditions. For instance, in developed studies about the antifungal action of EOs against the anthracnose pathogens in tropical fruits, in vivo tests practically are not included (Kumar & Kudachikar, 2018). They are needed in order to evidence the individual and synergistic effects of HBVC more clearly and show their true effectiveness against the disease (Bautista–Baños et al., 2003; Nikkhah, Hashemi, Habibi Najafi, & Farhoosh, 2017).

For these investigations, the type of treatment and conditions must be clearly defined depending on the aims. To conceive a treatment against superficial contamination of the products, it is easier than to develop a treatment to avoid pathogens inside the fruit. For this, several physical barriers (plant cuticle, subcuticular cells, and pathogen membranes) and processes (fruit and pathogen metabolism, internal absorption mechanisms during translocation) have to be overcome. Therefore, integrated knowledge about mechanisms of pathogens and antimicrobial compounds action and respiration and transpiration processes in fruit and vegetables must be considered in order to conceive any research project it includes. More knowledge about the antimicrobial mechanisms of action of EOs and PPhs at the molecular level, rather than just at the cellular level is required (Basak & Guha, 2018; Gómez‐López, 2012; Rao et al., 2019; Romanazzi et al., 2016; Wenchao et al., 2019).

Natural alternatives would have a better chance of success if they have been applied at optimum timing, and thus, the early and rapid detection of pathogens is important. Consequently, smart innovative tools and technologies are needed to improve the accuracy and promptness in diagnosing plant pathogens and foodborne microorganisms and their toxic metabolites (Albonico, Schutz, Caloni, Cortinovis, & Spicer, 2017; Cortés‐Higareda et al., 2019). They will facilitate high‐throughput analysis and efficient monitoring (Sanzani et al., 2016).

Finally, it is necessary to consider the role of genomic knowledge which gives information about gene functions, molecular receptors, and metabolic reactions that allow a better understanding of the physiological changes in fruit and vegetables and favors the optimization of treatments to control postharvest decay (Droby et al., 2016; Fernandez‐Blanco, Frizzell, Shanon, Ruíz, & Connolly, 2016). On the other hand, recent progress in metagenomic technologies should be used to characterize the composition of microbiota on products and its dynamics (Droby et al., 2016). Thus, this information may be useful to develop a new paradigm in postharvest biocontrol and in strategies of use of natural antimicrobials also. Therefore, more knowledge about the antimicrobial mechanisms of action of EOs and PPhs at the molecular level, rather than just at the cellular level is required (Rao et al., 2019).

Besides that, considering the value of nanotechnology‐related combined preservation strategies and nanotechnology‐related intelligent labeling system, modeling studies, legal aspects, safety concerns, and technical optimization need to be taken into account in future researches (Kothalawala & Sivakumaran, 2018; Maes et al., 2019; Wenchao et al., 2019).

From the commercial point of view, it is recognized that the most important limitation related with natural antimicrobial agents is the registration process. Some components of EOs with antimicrobial activity are registered by USA as flavoring agents for foods (carvacrol, carvone, cinnamaldehyde, citral, p‐cymene, eugenol, limonene, menthol, and thymol), while others such as estragole and methyl eugenol are not allowed because of their genotoxicity (Mari et al., 2016). Then, there is a need to develop more detailed toxicological studies to evaluate the health risks of postharvest treatments and to understand the decomposition mechanisms of these substances in organism (Mari et al., 2016). Among other relevant aspects, the organoleptic properties of the fruit are very important since the taste should not be affected by the treatment or by other quality attributes, related to the appearance, the nutrient concentration, etc. (Mari et al., 2016; Palou et al., 2016).

Finally, besides the satisfactory results at laboratory or semi‐industrial level, a techno‐economic feasibility study it is indispensable, in order to glimpse the cost‐benefit relationship of the proposed production process. It will depend on infrastructure, raw material availability, local needs and technical facilities, among other factors. The integral approach, must be consider for costeffectiveness control programs of preharvest, harvest, and post‐harvest processes.

For successfully development of this technology will be indispensable to attend the cost and energy sustainability, applying strategies related with circular economy. Mass and energy integration are necessary for reducing the costs and environmental impact. The use of the biorefinery‐based platform concept will enhance the efficiency of the plant residues utilization and to develop technological and marketing approaches for production of other HBVC and bioenergy (Moncada, Tamayo, & Cardona, 2014). Recently, a biorefinery model for PPhs, ethanol, and xylitol coproduction from spent blackberry pulp was developed by Davila, Rosenberg, and Cardona (2017). According to the sale‐to‐total‐production‐cost ratio, they demonstrated the importance of the mass and heat integration. The productivity values of 193.4, 6,912, and 452.2 kg/day of polyphenols, ethanol, and xylitol and yields of 3.88, 352.9, and 18.37 kg/ton of spent blackberry pulp were obtained, respectively.

On the other hand, it is necessary to develop cheaper technological alternatives, on the basis of efficient emergent extraction methods, and mainly drying techniques used during the raw material pretreatment, extracts concentration, and encapsulates formation. In this sense, some studies about spray drying conditions were carried out in order to avoid high energy consumption. For instance, the best conditions for the energy required, production costs, and physicochemical characteristics of the cheese whey were determined by Domínguez‐Niño, Cantú‐Lozano, Ragazzo‐Sánchez, Andrade‐González, and Luna‐Solano (2018). A dried product of 0.2165 kg/hr was obtained, with a moisture content of 2.08%, cost of 17.06 $/kg, and energy consumption of 2.0490 kW·hr/kg of dry product. Besides that, great interest to use and optimization of the renewable energies for production processes has been observed (Costales, 2010).

## 9 CONCLUSIONS

The use of natural antimicrobial agents as EOs and PPhs is a more ecological alternative technology to the synthetic and chemical fungicides currently used to control pathogens in postharvest processes. Among emerging technologies, edible coatings, films, and encapsulation techniques are showing advances and ensure improvement of the treatment effectiveness.

However, there are still many limitations that make difficult the implementation of this technology as a control strategy. Among some, challenges must be overcome for the industrial application, is necessary to consider the variability in quantity and quality of natural products, depending on the source, extraction conditions, and characterization methods. It is needed to show their true effectiveness against the disease in order to evidence the individual and synergistic effects.

Concerning methods, smart innovative tools are needed to improve the accuracy and promptness in diagnosing plant pathogens, foodborne microorganisms, and their toxic metabolites. On the other hand, it is indispensable to increase experimental tests in vivo, in order to confirm the laboratory in vitro results, and to apply a holistic method of analysis of postharvest process for improving the efficiency of treatments. Finally, genomic knowledge will give information about gene functions, molecular receptors, and metabolic reactions, allowing a better understanding of the physiological changes in fruit and vegetables and favoring the optimization of treatments also. Besides that, more studies about the microbiome diversity and composition on harvested products and its variability after harvest and during storage are necessary.

Therefore, attention must be paid to their toxicological properties, mechanisms of action against the pathogens and the effect on the organoleptic properties of the products. All these aspects will favor the successful implementation and commercialization of natural antimicrobial postharvest technology, but a cost‐benefit relationship, toxicity and ecotoxicity analysis will be indispensable in order to comply the sustainability of this technology.

## CONFLICT OF INTEREST

The authors declare that they do not have any conflict of interest and written informed consent was obtained from all of them.

## ETHICAL STATEMENTS

This study does not involve any human or animal testing.

## References

[fsn31437-bib-0001] Albonico, M. , Schutz, J. F. , Caloni, F. , Cortinovis, C. , & Spicer, I. J. (2017). *In vitro* effects of the *Fusarium micotoxins* fumonism B1 and beauvericin on bovine granulosa cell proliferation and steroid production. Toxicon, 128, 38–45. 10.1016/j.toxicon.2017.01.019 28132864

[fsn31437-bib-0002] Al‐Mamoori, F. , & Al‐Janabi, R. (2018). Recent advances in microwave‐assisted extraction (MAE) of medicinal plants: A review. International Research Journal of Pharmacy, 9, 22–29. 10.7897/2230-8407.09684

[fsn31437-bib-0003] Aloui, H. , Khwaldia, K. , Sánchez‐González, L. , Muneret, L. , Jeandel, C. , Hamdi, M. , & Desobry, S. (2014). Alginate coatings containing grapefruit essential oil or grapefruit seed extract for grapes preservation. International Journal of Food Science & Technology, 49, 952–959. 10.1111/ijfs.12387

[fsn31437-bib-0004] Anaya‐Castro, M. A. , Ayala‐Zavala, J. F. , Muñoz‐Castellanos, L. , Hernández‐ Ochoa, L. , Peydecastaing, J. , & Durrieu, V. (2017). β‐Cyclodextrin inclusion complexes containing clove (*Eugenia caryophyllata*) and Mexican oregano (*Lippia berlandieri*) essential oils: Preparation, physicochemical and antimicrobial characterization. Food Packaging and Shelf Life, 14 (Part B), 96–101. 10.1016/j.fpsl.2017.09.002.

[fsn31437-bib-0005] Arias, C. J. , & Toledo, J. (2018). Manual de Manejo Postcosecha de Frutas Tropicales. Organización de las Naciones Unidas para la Agricultura y la Alimentación (FAO). http://www.fao.org/inpho/content/documents/vlibrary/ac304s/ac304s00.htm. Accessed April 26, 2018

[fsn31437-bib-0006] Ariyarathna, I. R. , & Karunaratne, D. N. (2016). Microencapsulation stabilizes curcumin for efficient delivery in food applications. Food Packaging and Shelf Life, 10, 79–86. 10.1016/j.fpsl.2016.10.005

[fsn31437-bib-0007] Ávila‐Sosa, R. , Hernández‐Zamoran, E. , López‐Mendoza, I. , Palou, E. , Jiménez Munguía, M. T. , Nevárez‐Moorillón, G. V. , & López‐Malo, A. (2012). Fungal inactivation by Mexican oregano (*Lippia berlandieri Schauer*) essential oil added to amaranth, chitosan, or starch edible films. Journal of Food Science, 75, 127–133. 10.1111/j.1750-3841.2010.01524.x 20492301

[fsn31437-bib-0008] Awolola, V. , Chenia, H. , Koorbanaly, N. , & Baijnath, H. (2014). Antibacterial and anti‐biofilm activity of flavonoids and triterpenes isolated from the extracts of *Ficus Sansibarica Warb*. Subsp. Sansibarica (Moraceae) Extracts. African Journal of Traditional Complementary & Alternative Medicines, 11(3), 124–131. 10.4314/ajtcam.v11i3.19 PMC4202430 25371574

[fsn31437-bib-0009] Azmir, J. , Zaidul, I. S. M. , Rahman, M. M. , Sharif, K. M. , Mohamed, A. , Sahena, F. , … Omar, A. K. M. (2013). Techniques for extraction of bioactive compounds from plant materials: A review. Journal of Food Engineering, 117, 426–436.

[fsn31437-bib-0010] Bakowska‐Barczak, A. M. , & Kolodziejczyk, P. P. (2011). Black currant polyphenols: Their storage stability and microencapsulation. Industrial Crops and Products, 34, 1301–1309. 10.1016/j.indcrop.2010.10.002

[fsn31437-bib-0011] Ballesteros, L. F. , Ramírez, M. J. , Orrego, C. E. , Teixeira, J. A. , & Mussatto, S. I. (2017). Encapsulation of antioxidant phenolic compounds extracted from spent coffee grounds by freeze‐drying and spray‐drying using different coating materials. Food Chemistry, 237, 623–631. 10.1016/j.foodchem.2017.05.142 28764044

[fsn31437-bib-0012] Barkai‐Golan, R. (2001). Attack mechanism of the pathogen In Postharvest diseases of fruit and vegetable development and control (Chapter 5, 54–65, pp. 418–419), Amsterdam: The Volcani Center, Bet‐Dagan, Israel, Amsterdam: Elsevier Science.

[fsn31437-bib-0013] Barrera, L. L. , & García, L. J. (2008). Actividad antifúngica de aceites esenciales y sus compuestos sobre el crecimiento de *Fusarium sp*. aislado de papaya (*Carica papaya*). Revista UDO Agrícola, 8, 33–41.

[fsn31437-bib-0014] Basak, S. , & Guha, P. (2018). A review on antifungal activity and mode of action of essential oils and their delivery as nano‐sized oil droplets in food system. Journal of Food Science and Technology, 55(12), 4701–4710. 10.1007/s13197-018-3394-5 30482966 PMC6233421

[fsn31437-bib-0015] Bautista‐Baños, S. (2006). El control biológico en la reducción de enfermedades postcosecha en productos hortofrutícolas: uso de microorganismos antagónicos. Revista Iberoamericana de Tecnología Postcosecha [en línea] 2006, 8 (julio): [Fecha de consulta: 12 de agosto de 2019] Disponible en: <http://www.redalyc.org/articulo.oa?xml:id=81380101> ISSN 1665‐0204.

[fsn31437-bib-0016] Bautista‐Baños, S. , Hernández‐López, M. , Bósquez‐Molina, E. , & Wilson, C. L. (2003). Effects of chitosan and plant extracts on growth of *Colletotrichum gloeosporioides*: Anthracnose levels and quality of papaya fruit. Crop Protection, 22, 1087–1092. 10.1016/S0261-2194(03)00117-0

[fsn31437-bib-0017] Bautista‐Baños, S. , Ramos‐García, M. L. , Hernández‐López, M. , Córdoba‐Alborez, L. , López‐Mora, L. I. , Gutiérrez‐Martínez, P. , & Sánchez, D. (2012). Use of scanning and transmission electron microscopy to identify morphological and cellular damage on phytopathogenic fungi due to natural products application In Méndez‐Vilas A. (Eds.) Current microscopy contributions to advances in science and technology (Edition I, pp. 401–405). Formatex Research Center.

[fsn31437-bib-0018] Borboa‐Flores, J. , Rueda‐Puente, E. O. , Acedo‐Félix, E. , Ponce, J. F. , Cruz‐Villegas, M. , García‐Hernández, J. L. , & Ortega‐Nieblas, M. M. (2010). Evaluación de la actividad antibacteriana *in vitro* de aceites esenciales contra *Clavibacter michiganensis* subespecie *michiganensis* . Tropical and Subtropical Agroecosystems, 12, 539–547.

[fsn31437-bib-0019] Bosquez‐Molina, E. , Bautista‐Baños, S. , & Morales‐López, J. (2009). Aceites esenciales: Bioconservadores con alto potencial en la industria alimentaria. Industria Alimentaria, 31, 12–24.

[fsn31437-bib-0020] Bosquez–Molina, E. , Ronquillo–de Jesús, E. , Bautista–Baños, S. , Verde–Calvo, J. R. , & Morales–López, J. (2010). Evaluation of the inhibitory effect of essential oils against *Colletotrichum gleosporioides* and *Rhizopus stolonifera* in stored papaya fruit and their possible application in coatings. Postharvest Biology and Technology, 57, 132–137. 10.1016/j.postharvbio.2010.03.008

[fsn31437-bib-0021] Calvo, J. , & Martínez‐Martínez, L. (2009). Mecanismos de acción de los antimicrobianos. Enfermedades Infecciosas y Microbiología Clínica, 27(1), 44–52. 10.1016/jeimc2008.11.001 19218003

[fsn31437-bib-0022] Çam, M. , İçyer, N. C. , & Erdoğan, F. (2014). Pomegranate peel phenolics: Microencapsulation, storage stability and potential ingredient for functional food development. Food Science and Technology, 55, 117–123. 10.1016/j.lwt.2013.09.011

[fsn31437-bib-0023] Campoy, S. , & Adrio, J. L. (2017). Antifungals. Biochemical Pharmacology, 133, 86–96. 10.1016/j.bcp.2016.11.019 27884742

[fsn31437-bib-0024] Chen, Q. , Hu, Z. , Yan‐Dong, F. , & Hanhua‐Liang, Y. (2016). Study of two‐stage microwave extraction of essential oil and pectin from pomelo peels. Food Science and Technology, 66, 538–545. 10.1016/j.lwt.2015.11.019

[fsn31437-bib-0025] Cortés‐Higareda, M. , Ramos‐García, M. L. , Correa‐Pacheco, Z. N. , & Del Río, J. C. (2019). Nanostructured chitosan/ propolis formulations: characterization and effect on the growth of *Aspergillus flavus* and production of aflatoxins. Heliyon, 5(5), e01776 10.1016/j.heliyon.2019.e01776 31193581 PMC6536732

[fsn31437-bib-0026] Costales, R. (2010). Aplicación de la energía renovable en el secado. Estado del arte y su potencial en las producciones agrícolas. Revista De Los Derivados De La Caña De Azúcar (ICIDCA), 44(2), 47–53.

[fsn31437-bib-0027] Cushnie, T. P. T. , & Lamb, A. J. (2005). Antimicrobial activity of flavonoids. International Journal of Antimicrobial Agents, 26, 343–356. 10.1016/j.ijantimicag.2005.09.002 16323269 PMC7127073

[fsn31437-bib-0028] Dávila, J. A. , Rosenberg, M. , & Cardona, C. A. (2017). A biorefinery for efficient processing and utilization of spent pulp of Colombian Andes Berry (*Rubus glaucus Benth*.): Experimental, techno‐economic and environmental assessment. Bioresource Technology, 223, 227–236. 27792932 10.1016/j.biortech.2016.10.050

[fsn31437-bib-0029] De la Vega, J. C. , Cañarejo, M. A. , & y Pinto, N.S., (2017). Avances en tecnología de atmósferas controladas y sus aplicaciones en la industria. Una Revisión. Información Tecnológica, 28(3), 75–81.

[fsn31437-bib-0030] Domínguez‐Niño, A. , Cantú‐Lozano, D. , Ragazzo‐Sánchez, J. A. , Andrade‐González, I. , & Luna‐Solano, G. (2018). Energy requirements and production cost of the spray drying process of cheese whey. Drying Technology, 597–608. 10.1080/07373937.2017.1350863

[fsn31437-bib-0031] Donsì, F. , & Ferrari, G. (2016). Esencial oil nanoemulsions as antimicrobial agents. Food of Biotechnology Journal, 233, 106–120. 10.1016/j.jbiotec.2016.07.005 27416793

[fsn31437-bib-0032] Droby, S. , Wisniewski, M. , Teixido, N. , Spadaro, D. , & Jijakli, M. H. (2016). The science, development and commercialization of postharvest biocontrol products. Postharvest Biology and Technology, 22–29, 10.1016/j.postharvbio.2016.04.00609

[fsn31437-bib-0033] Drosou, C. G. , Magdalini, K. , Krokida, C. , & Biliaderis, G. (2010). Encapsulation of bioactive compounds through electrospinning/ electrospraying and spray drying: A comparative assessment of food‐related applications. Drying Technology, 35, 139–162. 10.1080/07373937.2016.1162797

[fsn31437-bib-0034] Eguillor, P. M. R. (2019). Pérdida y desperdicio de alimentos en el sector agrícola: avances y desafíos. Edición de Oficina de Estudios y Políticas Agrarias –ODEPA. www.odepa.gob.cl

[fsn31437-bib-0035] Esfanjani, A. F. , & Jafari, S. M. (2016). Biopolymer nano‐particles and natural nano‐carriers for nano‐encapsulation of phenolic compounds. Colloids and Surfaces B: Biointerfaces, 146, 532–543. 10.1016/j.colsurfb.2016.06.053. 27419648

[fsn31437-bib-0036] Espina, L. , Somolinos, M. , Lorán, S. , Conchello, P. , García, D. , & Pagán, R. (2011). Chemical composition of commercial citrus fruit essential oils and evaluation of their antimicrobial activity acting alone or in combined processes. Food Control, 22, 896–902. 10.1016/j.foodcont.2010.11.021.

[fsn31437-bib-0037] Ezhilarasi, P. N. , Karthik, P. , Chhanwal, N. , & Anandharamakrishnan, C. (2013). Nanoencapsulation techniques for food bioactive components: A Review. Food and Bioprocess Technology, 6, 628–647. 10.1007/s11947-012-0944-0.

[fsn31437-bib-0038] Fagundes, C. , Pérez‐Gago, M. B. , Monteiro, A. R. , & Palou, L. (2013). Antifungal activity of food additives in vitro and as ingredients of hydroxypropyl methylcellulose‐lipid edible coatings against Botrytis cinerea and Alternaria alternata on cherry tomato fruit. International Journal of Food, 166, 391–398. 10.1016/j.ijfoodmicro.2013.08.001. 24026010

[fsn31437-bib-0039] FAO (2019). El sistema alimentario en México Oportunidades para el campo mexicano en la Agenda 2030 de Desarrollo Sostenible. Organización de Naciones Unidas para la Alimentación y la Agricultura.

[fsn31437-bib-0040] Fathi, M. , Martín, A. , & McClements, D. J. (2014). Nanoencapsulation of food ingredients using carbohydrate based delivery systems. Trends in Food Science and Technology, 39, 18–39.

[fsn31437-bib-0041] Fernández, N. M. , Echeverria, D. C. , Mosquera, S. A. , & Paz, S. P. (2017). Estado actual del uso de recubrimientos comestibles en frutas y hortalizas. *Biotecnología en el Sector Agropecuario y* . Agroindustrial, 15(2), 134–141. 10.18684/BSAA.

[fsn31437-bib-0042] Fernández, X. , Minuti, M. , Visinoni, F. , Cravotto, G. , & Chemat, F. (2013). Solvent‐free microwave extraction of essential oil from aromatic herbs: From laboratory to pilot and industrial scale. Food Chemistry, 150(1), 193–198. 10.1016/j.foodchem.2013.10.139 24360439

[fsn31437-bib-0043] Fernández‐Blanco, C. , Frizzell, C. , Shanon, M. , Ruíz, M. J. , & Connolly, L. (2016). An *in vitro* investigation on the cytotoxic and nuclear receptor transcriptional activity of the mycotoxins fumonism B1 and beauvericin. Toxicology Letters, 257, 1–10. 10.1016/j.toxlet.2016.05.021 27234500

[fsn31437-bib-0044] Fisher, K. , & Phillips, C. (2008). Potencial antimicrobial uses of essential oils in food: Is citrus the answer? Trends in Food Science & Technology, 19, 156–164. 10.1016/j.tifs.2007.11.006

[fsn31437-bib-0045] Franco‐Vega, A. , Palou, E. , & López‐Malo, A. (2012). Combinación de ultrasonido de baja frecuencia con factores convencionales y/o emergentes como métodos de inactivación de microorganismos en alimentos. Temas Selectos En Ingeniería En Alimentos, 6, 73–83.

[fsn31437-bib-0046] García‐Robles, J. M. , Medina‐Rodríguez, L. J. , Mercado‐Ruiz, J. N. , & Báez‐Sañudo, R. (2017). Evaluación de desinfectantes para el control de microorganismos en frutas y verduras. Revista Iberoamericana De Tecnología Postcosecha, 18, 9–22.

[fsn31437-bib-0047] Gómez‐López, V. (2012). Decontamination of fresh and minimally processed produce. Hoboken, NJ: Wiley‐Blackwell, A John Wiley & Sons Ltd, Publication.

[fsn31437-bib-0048] Gómez‐Mascaraque, L. G. , Hernandez‐Rojas, M. , Tarancon, P. , Tenon, M. , Gong, L. , Li, T. , … Jiang, Y. (2016). An inclusion complex of eugenol into β‐cyclodextrin: Preparation, and physicochemical and antifungal characterization. Food Chemistry, 196, 324–330. 10.1016/j.foodchem.2015.09.052 26593497

[fsn31437-bib-0049] Gong, L. , Li, T. , & Chen, F., Duan, X. , Yuan, Y. , Zhang, D. , Jiang, Y. (2016). An inclusion complex of eugenol into β-cyclodextrin: Preparation, and physicochemical and antifungal characterization. Food Chemistry, 196, 324–330. 26593497 10.1016/j.foodchem.2015.09.052

[fsn31437-bib-0050] González‐Estrada, R. , Carvajal‐Millán, E. , Ragazzo‐Sánchez, J. A. , Bautista‐Rosales, P. U. , & Calderón‐Santoyo, M. (2017). Control of blue mold decay on *Persian lime*: Application of covalently cross‐linked arabinoxylans bioactive coatings with antagonistic yeast entrapped. Food Science and Technology, 85, 187–196. 10.1016/j.lwt.2017.07.019

[fsn31437-bib-0051] Guarda, J. F. , Rubilar, J. , Miltz, M. , & Galotto, J. (2011). The antimicrobial activity of microencapsulated thymol and carvacrol. International Journal of Food and Microbiology, 146, 144–150. 10.1016/j.ijfoodmicro.2011.02.011 21411168

[fsn31437-bib-0052] Hernández, J. A. , Torres‐Chávez, P. I. , Ramírez‐Wong, B. , Ráscon‐Chu, A. , Plascencia‐Jatomea, M. , Barreras‐Urbina, C. G. , … Rodríguez‐Félix, F. (2015). Micro‐ and nanoparticles by electrospray: Advances and applications in foods. Journal of Agricultural and Food Chemistry, 63, 4699–4707. 10.1021/acs.jafc.5b01403 25938374

[fsn31437-bib-0053] Jagtap, U. B. , & Bapat, V. A. (2010). *Artocarpus*: A review of its traditional uses, phytochemistry and pharmacology. Review. Journal of Ethnopharmacology, 129, 142–166. 10.1016/j.jep.2010.03.031 20380874

[fsn31437-bib-0054] Kamil, C. , Chen, O. , & Blumberg, J. (2015). The application of nanoencapsulation to enhance the bioavailability and distribution of polyphenols In Sabilov C., Chen H., & Yada R. (Eds.), Nanotechnology and fuctional foods ‐ Effective delivery of bioactive ingredients (pp. 158‐164). USA: IFT Press.

[fsn31437-bib-0055] Khan, M. A. , Rahman, A. A. , Islam, S. , Khandokhar, P. , Parvin, S. , Islam, M. B. , … Alam, H. M. K. (2013). A comparative study on the antioxidant activity of methanolic extracts from different parts of *Morus alba* L. (*Moraceae)*. *BMC Research* . Notes, 6, 24 10.1186/1756-0500-6-24 PMC3559264 23331970

[fsn31437-bib-0056] Kothalawala, S. G. , & Sivakumaran, K. (2018). An overview of nanotechnology applications in food industry. International Journal of Scientific and Research Publications, 8, 340–343. 10.29322/IJSRP.8.7.2018.p7954.

[fsn31437-bib-0057] Kumar, A. , & Kudachikar, V. B. (2018). Antifungal properties of essential oils against anthracnose disease: A critical appraisal. Journal of Plant Diseases and Protection, 125, 133–144. 10.1007/s41348-017-0128

[fsn31437-bib-0058] Kumari, S. , Pundhir, S. , Priya, P. , Jeena, G. , Punetha, A. , Chawla, K. , … Yadav, G. (2014). EssOilDB: a database of essential oils reflecting terpene composition and variability in the plant kingdom, 120. 10.1093/database/bau120 PMC4273207 25534749

[fsn31437-bib-0059] Leyva, M. , Mounier, J. , Valence, F. , Coton, M. , Thierry, A. , & Coton, E. (2017). Antifungal microbial agents for food biopreservation ‐ A review. Microorganisms, 5(3), 37 10.3390/microorganisms5030037 PMC5620628 28698479

[fsn31437-bib-0060] Liang, J. , Yan, H. , Puligundla, P. , Gao, X. , Zhou, Y. , & Wan, X. (2017). Applications of chitosan nanoparticles to enhance absorption and bioavailability of tea polyphenols: A review. Food Hydrocolloids, 69, 286–292. 10.1016/j.foodhyd.2017.01.041

[fsn31437-bib-0061] Liu, M. , Zheng, Y. , Wang, C. , Xie, J. , Wang, B. , Wang, Z. , … Ni, M. (2016). Improved stability of (+)‐catechin and (−)‐epicatechin by complexing with hydroxypropyl‐β‐cyclodextrin: Effect of pH, temperature and configuration. Food Chemistry, 196, 148–154. 10.1016/j.foodchem.2015.09.016 26593476

[fsn31437-bib-0062] Loizzo, M. R. , Tundis, R. , Chandrika, U. G. , Abeysekera, A. M. , Menichini, F. , & Frega, N. G. (2010). Antioxidant and antibacterial activities on foodborne pathogens of *Artocarpus heterophyllus* Lam. (*Moraceae*) leaves extracts. Journal of Food Science, 75, 291–295. 10.1111/j.1750-3841.2010.01614.x 20629886

[fsn31437-bib-0063] Maes, C. , Bouquillon, S. , & Fauconnier, M. L. (2019). Encapsulation of essential oils for the development of biosourced pesticides with controlled release: A Review. Molecules, 24, 2539 10.3390/molecules24142539 PMC6680563 31336803

[fsn31437-bib-0064] Manchón, N. (2013). Métodos avanzados para el análisis, conservación y extracción de compuestos fenólicos en alimentos. Tesis presentada por para optar al grado de Doctora por la Universidad de Valladolid, Facultad de Ciencias, Departamento de Química Analítica.

[fsn31437-bib-0065] Mari, M. , Bautista‐Baños, S. , & Sivakumar, D. (2016). Decay control in the postharvest system: Role of microbial and plant volatile organic compounds. Postharvest. Biology and Technology, 122, 70–81. 10.1016/j.postharvbio.2016.04.014

[fsn31437-bib-0066] Marín, A. , Atarés, L. , & Chiralt, A. (2017). Improving function of biocontrol agents incorporated in antifungal fruit coatings: A review. Biocontrol Science and Technology, 2, 23–28. 10.1080/09583157.2017.1390068

[fsn31437-bib-0067] Meireles, A. , Giaouris, E. , & Simies, M. (2016). Alternative disinfection methods to chlorine for use in the fresh‐cut industry. Food Research International, 82, 71–85. 10.1016/j.foodres.2016.01.021

[fsn31437-bib-0068] Melgarejo, J. G. , & Postilla, A. (2011). Fungicidas‐ mecanismos de acción de los fungicidas, en “Manejo Integrado de Enfermedades”, 11, 193–202.

[fsn31437-bib-0069] Moncada, J. , Tamayo, J. A. , & Cardona, C. A. (2014). Integrating first, second, and third generation biorefineries: Incorporating microalgae into the sugarcane biorefinery. Chemical Engineering Science, 118, 126–140.

[fsn31437-bib-0070] Nikkhah, M. , Hashemi, M. , Habibi Najafi, M. B. , & Farhoosh, R. (2017). Synergistic effects of some essential oils against fungal spoilage on pear fruit. International Journal of Food Microbiology, 18, 257:285–294. 10.1016/j.ijfoodmicro.2017.06.021. 28763743

[fsn31437-bib-0071] Ochoa‐Fuentes, Y. M. , Cerna‐ Chávez, E. , Landeros‐Flores, J. , Hernández‐Camacho, S. , & y Delgado‐Ortiz, J.C., (2012). Evaluación *in vitro* de la actividad antifúngica de cuatro extractos vegetales metanólicos para el control de tres especies de *Fusarium spp* . Revista Internacional De Botánica Experimental, 81(1), 69–73.

[fsn31437-bib-0072] Ojeda‐Contreras, A. J. , Hernández‐Martínez, J. , Domínguez, Z. , & Mercado, J. (2008). Utilization of caffeic acid phenethyl ester to control *Alternaria alternata* rot in tomato (*Lycopersicon esculentum Mill.)* Fruit. Journal of Phytopathology, 156, 164–173. 10.1111/j.1439-0434.2007.01325.x

[fsn31437-bib-0073] Ojwang, R. A. , Muge, E. K. , Mbatia, B. , Mwanza, B. , & Ogoyi, D. (2017). Comparative analysis roots and of phytochemical composition and antioxidant activities of methanolic extracts of leaves, bark of jackfruit (*Artocapus heterophyllus*) from selected regions in Kenya and Uganda. Journal of Advances in Biology & Biotechnology, 16(1), 1–13. 10.9734/EJMP/2018/40967

[fsn31437-bib-0074] Oliveira, D. M. , Ferreira, R. S. , & Gomes, C. L. (2017). Nanoencapsulation of passion fruit byproducts extracts for enhanced antimicrobial activity. Food and Bioproducts Processing, 104, 137–146. 10.1016/j.fbp.2017.05.0090960-3085

[fsn31437-bib-0075] Pagnussatt, F. A. , Kupski, L. , Darley, F. T. , Filoda, P. F. , Ponte, É. M. D. , Garda‐Buffon, J. , & Badiale‐Furlong, E. (2013). *Fusarium graminearum* growth inhibition mechanism using phenolic compounds from *Spirulina sp* . Ciência E Tecnologia De Alimentos, 33, 75–80. 10.1590/s0101-20612013000500012

[fsn31437-bib-0076] Palou, L. , Ali, A. , Fallik, E. , & Romanazzi, G. (2016). GRAS, plant‐ and animal‐derived compounds as alternatives to conventional fungicides for the control of postharvest diseases of fresh horticultural produce. Postharvest Biology and Technology, 122, 41–52. 10.1016/j.postharvbio.2016.04.01

[fsn31437-bib-0077] Parafati, M. , Vitale, A. , Resstucia, C. , & Cirvirelly, G. (2016). The effect of locust bean gum (LBG)‐based edible coatings carrying biocontrol yeast against *Penicillium digitatum* and *Penicillium italicum* causal agents of postharvest decay of mandarin fruit. Food Microbiology, 58, 87–94. 10.1016/j.fm.2016.03.014 27217363

[fsn31437-bib-0078] Paredes‐López, O. , Cervantes‐Ceja, M. L. , Vigna‐Pérez, M. , & Hernández‐Pérez, T. (2010). Berries: Improving human health and healthy aging, and promoting quality life‐ a review. Plant Foods Human Nutrition, 65, 299–308. 10.1007/s11130-010-0177-1 20645129

[fsn31437-bib-0079] Pekmezovic, M. , Rajkovic, K. , Barac, A. , Senerovic, L. , & Arsic, A. V. (2015). Development of kinetic model for testing antifungal effect of *T. vulgaris* L. and *Cinnamomum cassia* L. essential oils on *Aspergillus flavus* spores and application for optimization of synergistic effect. Biochemical Engineering Journal, 99, 131–137. 10.1016/j.bej.2015.03.024.

[fsn31437-bib-0080] Pereira, M. , Hill, I. , & Zambiazi, R. (2015). Nanoencapsulation of hydrophobic phytochemicals using poly (dl‐lactide‐co‐glycolide) (PLGA) for antioxidant and antimicrobial delivery applications: Guabiroba fruit (*Campomanesia xanthocarpa* O. Berg) study. Food Science and Technology, 63, 100–107. 10.1016/j.lwt.2015.03.062

[fsn31437-bib-0081] Pereira, M. C. , Oliveira, D. A. , Hill, L. E. , Rui, C. , Zambiazi, C. D. , Borges, M. , … Gomes, L. (2018). Effect of nanoencapsulation using PLGA on antioxidant and antimicrobial activities of Guabiroba fruit phenolic extract. Food Chemistry, 240, 396–404. 10.1016/j.foodchem.2017.07.14 28946289

[fsn31437-bib-0082] Pino, O. , Sánchez, I. , & Rojas, M. M. (2013). Plant secondary metabolites as alternatives in pest management. II: An overview of their potential. Revista De Protección Vegetal (online), 28, 95–108.

[fsn31437-bib-0083] Ponce, A. , Roura, S. , del Valle, C. , & Moreira, Μ. (2008). Antimicrobial and antioxidant activities of edible coatings enriched with natural plant extracts: *In vitro and in vivo* studies. Postharvest Biology and Technology, 49, 294–300. 10.1016/j.postharvbio.2008.02.013

[fsn31437-bib-0084] Ramírez‐Reyes, T. , Flores‐Estévez, N. , Luna‐Rodríguez, M. , Noa‐Carrazana, J. C. , Sánchez‐Velásquez, L. R. , & Trigos‐Landa, A. (2015). Extractos crudos de *Magnolia schiedeana* Schltdl. para el control de bacterias fitopatógenas. Madera y Bosques, 21, 159–164.

[fsn31437-bib-0085] Ramos–García, L. M. , Bautista‐Baños, S. , & Estrada–Carrillo, M. (2010). Compuestos antimicrobianos adicionados en recubrimientos comestibles para uso en productos hortofrutícolas. Revista Mexicana De Fitopatología, 28, 44–57.

[fsn31437-bib-0086] Rao, J. , Chen, B. , & McClement, D. J. (2019). Improving the efficacy of essential oils as antimicrobials in food: Mechanism of action. Annual Review Food, 10, 3.1‐3.23 10.1146/annurev-food-032818-121727 30653350

[fsn31437-bib-0087] Regulation (EU) No 528/2012 . Regulation (EU) No 528/2012 of the European Parliament and of the Council of 22 May 2012 concerning the making available on the market and use of biocidal products, Text with EEA relevance.

[fsn31437-bib-0088] Ribeiro‐Santos, Y. R. , & Rezende Pedreira‐Nogueira, J. (2018). Microencapsulation of extracts of bioactive compounds obtained from acerola (*Malpighia emarginata* DC) pulp and residue by spray and freeze drying: Chemical, morphological and chemometric characterization. Food Chemistry, 254, 281–291. 10.1016/j.foodchem.2018.02.026 29548455

[fsn31437-bib-0089] Rodríguez, J. , Martín, M. J. , Ruiz, M. A. , & Clares, B. (2016). Current encapsulation strategies for bioactive oils: From alimentary to pharmaceutical perspectives. Food Research International, 83, 41–59. 10.1016/j.foodres.2016.01.032

[fsn31437-bib-0090] Rodríguez‐García, I. , Silva‐Espinoza, B. A. , Ortega‐Ramírez, L. A. , Leyva, J. M. , Siddiqui, M. W. , Cruz‐Valenzuela, M. R. , … Ayala‐Zavala, J. F. (2016). Oregano Essential Oil as an Antimicrobial and Antioxidant Additive in Food Products. Critical Reviews in Food Science and Nutrition, 1717–1727, 10.1080/10408398.2013.800832 25763467

[fsn31437-bib-0091] Rodríguez‐Maturino, A. , Troncoso‐Rojas, R. , Sánchez‐Estrada, A. , González‐Mendoza, D. , Ruiz‐Sánchez, E. , Zamora‐Bustillos, R. , … Avilés‐Marina, M. (2015). Efecto antifúngico de extractos fenólicos y de carotenoides de chiltepín (*Capsicum annum* var. glabriusculum) en *Alternaria alternata* y *Fusarium oxysporum* . Revista Argentina De Microbiología, 47, 72–77. 10.1016/j.ram.2014.12.005 25705046

[fsn31437-bib-0092] Rojas, M. M. , Corzo, M. , Sánchez, Y. , Brito, D. , Montes de Oca, R. , Martínez, Y. , & y Pino, O. (2014). Actividad antibacteriana de aceites esenciales sobre *Pectobacterium carotovorum* subsp. *carotovorum* . Revista De Protección Vegetal, 29,197‐203.

[fsn31437-bib-0093] Romanazzi, G. , Sanzani, S. M. , Bi, Y. T. S. , Martínez, P. G. , & Alkan, N. (2016). Induced resistance to control postharvest decay of fruit and vegetables. Postharvest Biology and Technology, 122, 82–94. 10.1016/j.postharvbio.2016.08.003

[fsn31437-bib-0094] Ruiz‐Barbai, R. M. , Ríos‐Sánchez, C. , Fedriani‐Irisol, J. M. , Oliasi, J. L. , & Rios, l., Jiménez‐Díaz, R., (1990). Bactericidal effect of phenolic compounds from green olives on *Lactobacillus plantarum*. L. Systematic and Applied Microbiology, 13, 199–205.

[fsn31437-bib-0095] Salem, M. Z. M. , Salem, A. Z. M. , Camacho, L. M. , & Hayssam, M. A. (2013). African Journal of Microbiology Antimicrobial activities and phytochemical composition of extracts of Ficus species: An overview. African Journal of Microbiology Research, 7, 4207–4219. 10.5897/AJMR2013.5570

[fsn31437-bib-0096] Sánchez, Y. , Correa, T. M. , & Y. y Pino, O., (2012a). Efecto del aceite esencial de *Piper marginatum* Jacq. y sus componentes sobre *Xanthomonas albilineans* (Ashby) Dawson Dowson y *Xanthomonas campestris* pv. *Campestris* (Pammel) Dowson. Revista De Protección Vegetal, 27, 39–44.

[fsn31437-bib-0097] Sánchez, Y. , Pino, O. , Rojas, M. M. , Sánchez, Y. , Abreu, Y. , Espinosa, I. , … y Pino, O., (2012b). Caracterización química y actividad antibacteriana de aceites esenciales de *Ocimum basilicum* L. y *Ocimum basilicum var. genovese* L. Revista De Protección Vegetal, 27(2), 130–134.

[fsn31437-bib-0098] Sandoval‐Contreras, T. , Villarruel‐ López, A. , Sierra‐ Beltrán, P. , & Torres‐Vitela, M. R. (2017). Effect of pH and temperature in production of mycotoxins and antibiotics by phytopathogenic moulds for Persian lime (*Citrus latifolia* T.) in a complex lime pericarp‐base medium. Emirates Journal of Food and Agriculture, 29, 751–759. 10.9755/ejfa.2017.v29.i10.1293

[fsn31437-bib-0099] Sanzani, S. M. , Reverberi, M. , & Geisen, R. (2016). Mycotoxins in harvested fruit and vegetable: Insights in producing fungi, biological role, conducive conditions, and tools to manage postharvest contamination. Postharvest Biology and Technology, 122, 95–105. 10.1016/j.postharvbio.2016.07.003

[fsn31437-bib-0100] Sarkar, D. , & Shetty, K. (2014). Metabolic stimulation of plant phenolics for food preservation and health. *Annual Review of* . Food Science and Technology, 5, 19.1–19.19 10.1146/annurev-food-030713-092418 24422591

[fsn31437-bib-0101] Shaaban, H. A. , El‐Ghorab, A. H. , & Shibamoto, T. (2012). Bioactivity of essential oils and their volatile aroma components: Review. Journal of Essential Oil Research, 24(2), 203–212. 10.1080/10412905.2012.659528

[fsn31437-bib-0102] Shishir, M. R. I. , Xie, L. , Sun, C. , Zheng, X. , & Chen, W. (2018). Advances in micro and nano‐encapsulation of bioactive compounds using biopolymer and lipid‐based transporters. Trends in Food Science & Technology, 78, 34–60. 10.1016/j.tifs.2018.05.018

[fsn31437-bib-0103] Silva, L. , Hill, L. , Figueredo, E. , & Gomes, C. (2014). Delivery of phytochemicals of tropical fruit by‐products using poly (dl‐lactide‐co‐glycolide) (PLGA) nanoparticles: Synthesis, characterization, and antimicrobial. Food Chemistry, 165, 362–370. 10.1016/j.foodchem.2014.05.118 25038688

[fsn31437-bib-0104] Sivakumar, D. , & Bautista‐Baños, S. (2014). A review on the use of essential oils for postharvest decay control y maintenance of fruit quality during storage. Crop Protection, 64, 27–37. 10.1016/j.cropro.2014.05.012

[fsn31437-bib-0105] Solís‐Silva, A. , Reyes‐Munguía, A. , Madariaga‐Navarrete, G. , Medina‐Pérez, R. G. , Campos‐Montiel, A. J. , & Cenobio‐Galindo, J. (2018). Evaluación de la actividad antifúngica y antioxidante de una nanoemulsión W/O de *Opuntia oligacantha* y aceite esencial de *Citrus X sinensis* . Investigación y Desarrollo en Ciencia Y Tecnología de Alimentos, 3, 182–187. 10.1016/jfoodchem2014.05.118

[fsn31437-bib-0106] Torrenegra, M. E. , Nerlis, A. , Pájaro, P. , & León‐Méndez, G. (2017). Actividad antibacteriana *in vitro* de aceites esenciales de diferentes especies del género Citrus. Revista Colombiana de Ciencias Químico‐Farmacéuticas, 46(2), 160–175. 10.15446/rcciquifa.v46n2.67934.

[fsn31437-bib-0107] Tripathi, P. , Dubey, N. K. , & Shukla, A. K. (2008). Use of some essential oils as post‐harvest botanical fungicides in the management of grey mould of grapes caused by *Botrytis cinerea* . World Journal of Microbiology and Biotechnology, 24, 39–46. 10.1007/s11274-007-9435-2

[fsn31437-bib-0108] Troncoso‐Rojas, R. , Espinoza, C. , Sánchez‐Estrada, A. , Tiznado, M. E. , García, H. , Turek, C. , & Stintzing, F. C. (2013). Stability of Essential Oils: A Review. Comprehensive Reviews in Food Science and Food Safety, 12, 213–220. 10.1111/1541-4337.12006

[fsn31437-bib-0109] Usman, H. , Abdulrahman, F. , & Usman, A. (2009). Qualitative phytochemical screening and *in vitro* antimicrobial effects of methanol stem bark extract of *Ficus thonningii* (*Moraceae*) *African Journal of Traditional* . Complementary and Alternative Medicine, 6, 289–295. 10.4314/ajtcam.v6i3.57178 PMC2816454 20448855

[fsn31437-bib-0110] Wenchao, L. , Zhang, M. , & Bhandari, B. (2019). Nanotechnology – A shelf life extension strategy for fruit and vegetable. Critical Reviews in Food Science and Nutrition, 1–16. 10.1080/10408398.2019.1589415 30896257

[fsn31437-bib-0111] Yilmaz, A. , Ermis, E. , & Boyraz, N. (2016). Investigation of *in vitro* and *in vivo* antifungal activities of different plant essential oils against postharvest apple rot deseases *Colletotrichum gleosporioides, Botrytis cinerea and Penicillium expansum* . Archiv für Lebensmittelhygiene, 67, 122–131. 10.2376/0003-925X-67-122

[fsn31437-bib-0112] Yu, H. , Park, J. Y. , Kwon, C. W. , Hong, S. C. , Park, K. M. , & Chang, P. S. (2018). An overview of nanotechnology in food science: preparative methods, practical applications, and safety. Journal of Chemistry, Article ID 5427978, 10 p. 10.1155/2018/5427978.

[fsn31437-bib-0113] Yuan, G. , Chen, X. , & Li, D. (2016). Chitosan films and coatings containing essential oils: The antioxidant and antimicrobial activity, and application in food systems. Food Research International, 89, 117–128. 10.1016/j.foodres.2016.10.004 28460897

[fsn31437-bib-0114] Zhao, X. , & Drlica, K. (2014). Reactive oxygen species and the bacterial response to lethal stress. Current Opinion in Microbiology, 21, 1–6. 10.1016/j.mib.2014.06.008 25078317 PMC4254325

